# Compare and Contrast Meta Analysis (CCMA): A Method for Identification of Pleiotropic Loci in Genome-Wide Association Studies

**DOI:** 10.1371/journal.pone.0154872

**Published:** 2016-05-05

**Authors:** Hansjörg Baurecht, Melanie Hotze, Elke Rodríguez, Judith Manz, Stephan Weidinger, Heather J. Cordell, Thomas Augustin, Konstantin Strauch

**Affiliations:** 1 Department of Dermatology, Allergology and Venereology, University Hospital Schleswig-Holstein, Campus Kiel, Kiel, Germany; 2 Research Unit of Molecular Epidemiology, Institute of Epidemiology II, Helmholtz Zentrum München - German Research Center for Environmental Health, Neuherberg, Germany; 3 Institute of Genetic Medicine, Newcastle University, Newcastle upon Tyne, United Kingdom; 4 Department of Statistics, Ludwig-Maximilians-Universität Munich, Munich, Germany; 5 Institute of Genetic Epidemiology, Helmholtz Zentrum München - German Research Center for Environmental Health, Neuherberg, Germany; 6 Institute of Medical Informatics, Biometry and Epidemiology, Chair of Genetic Epidemiology, Ludwig-Maximilians-Universität, Munich, Germany; University of Texas School of Public Health, UNITED STATES

## Abstract

In recent years, genome-wide association studies (GWAS) have identified many loci that are shared among common disorders and this has raised interest in pleiotropy. For performing appropriate analysis, several methods have been proposed, e.g. conducting a look-up in external sources or exploiting GWAS results by meta-analysis based methods. We recently proposed the Compare & Contrast Meta-Analysis (CCMA) approach where significance thresholds were obtained by simulation. Here we present analytical formulae for the density and cumulative distribution function of the CCMA test statistic under the null hypothesis of no pleiotropy and no association, which, conveniently for practical reasons, turns out to be exponentially distributed. This allows researchers to apply the CCMA method without having to rely on simulations. Finally, we show that CCMA demonstrates power to detect disease-specific, agonistic and antagonistic loci comparable to the frequently used Subset-Based Meta-Analysis approach, while better controlling the type I error rate.

## Introduction

Genome-wide association studies (GWAS) have identified many loci that are shared among common disorders. [1] The interest in pleiotropy, “the multi-functionality of a gene in phenotype presentation”, [2] has increased in recent years. Customized arrays have been designed by consortia of related diseases (e.g. the Immunochip array for immune-mediated disorders), to fine map established GWAS loci at high resolution and identify single nucleotide variants (SNVs) shared among different traits.

For performing an appropriate analysis, several methods [1, 2] have been proposed that use external sources such as the GWAS catalog. [3] Others exploit GWAS results using meta-analysis based methods. [4, 5] We have recently proposed the Compare & Contrast Meta-Analysis (CCMA) approach [6] and have found suitable P-value thresholds corresponding to standard suggestive (*P* < 10^−5^) and genome wide significant (*P* < 10^−8^) association by simulation. In this work we present an analytical cumulative distribution function for the CCMA test statistic, which is in good accordance with the levels derived by simulation studies.

## Materials and Methods

As we previously described [6], the CCMA uses z-scores from GWAS of two different traits, *T*_1_ and *T*_2_, which are asymptotically normally distributed and signed according to the direction of effect of a certain reference allele. Furthermore, two z-scores for meta analysis are defined, assuming an agonistic or an antagonistic action of the variant on the two traits [6]. Then the CCMA test statistic is constructed as

$$\begin{array}{c} T_{max}=\max(|T_{1}\left|,\right|T_{2}\left|,\right|T_{\text{12,agonistic}}\left|,\right|T_{\text{12,antagonistic}}\left|\right) \end{array}$$ (1)

where

$$\begin{array}{ccc} T_{\text{12,agonistic}} & = & \frac{T_{1}+T_{2}}{\sqrt{2}}\;\text{and}\;\\ T_{\text{12,antagonistic}} & = & \frac{T_{1}-T_{2}}{\sqrt{2}}. \end{array}$$

In order to derive a P-value for an observed realization *t*_*max*_, the null distribution was empirically determined by simulating *R* = 1,000,000,000 replicates of two normally distributed random variables *Z*_1_ and *Z*_2_. Then $Z_{\text{12,agonistic}}=\frac{Z_{1}+Z_{2}}{\sqrt{2}}$, $Z_{\text{12,antagonistic}}=\frac{Z_{1}-Z_{2}}{\sqrt{2}}$ and

$$\begin{array}{c} Z_{max}=\max(|Z_{1}\left|,\right|Z_{2}\left|,\right|Z_{\text{12,agonistic}}\left|,\right|Z_{\text{12,antagonistic}}\left|\right) \end{array}$$ (2)

was calculated for each replicate. The empirical P-values can be derived as

$$\begin{array}{c} P_{emp}=\frac{\#(Z_{max}>t_{max})+1}{R+1} \end{array}$$

In order to find an analytic formulation of the P-value distribution we consider the squared values of the test statistics $Z_{1}^{2},Z_{2}^{2},Z_{\text{12,agonistic}}^{2},Z_{\text{12,antagonistic}}^{2}$ under the null hypothesis (H_0_) of no pleiotropy and no association between the SNV and any trait. By design, each of the four transformed variables follows a $\chi_{1}^{2}$ distribution with $Z_{1}^{2}⊥Z_{2}^{2}$ and $Z_{\text{12,agonistic}}^{2}⊥Z_{\text{12,antagonistic}}^{2}$ under H_0_ (see S1 Appendix). Thus, the transformed CCMA test statistic can be expressed as

$$\begin{array}{c} Z_{max}^{2}=\max\left(Z_{1}^{2},Z_{2}^{2},Z_{\text{12,agonistic}}^{2},Z_{\text{12,antagonistic}}^{2}\right) \end{array}$$ (3)

and empirical P-values can be calculated for an observed realization by

$$\begin{array}{c} P_{emp}=\frac{\#(Z_{max}^{2}>t_{max}^{2})+1}{R+1} \end{array}$$ (4)

**Fig 1:**
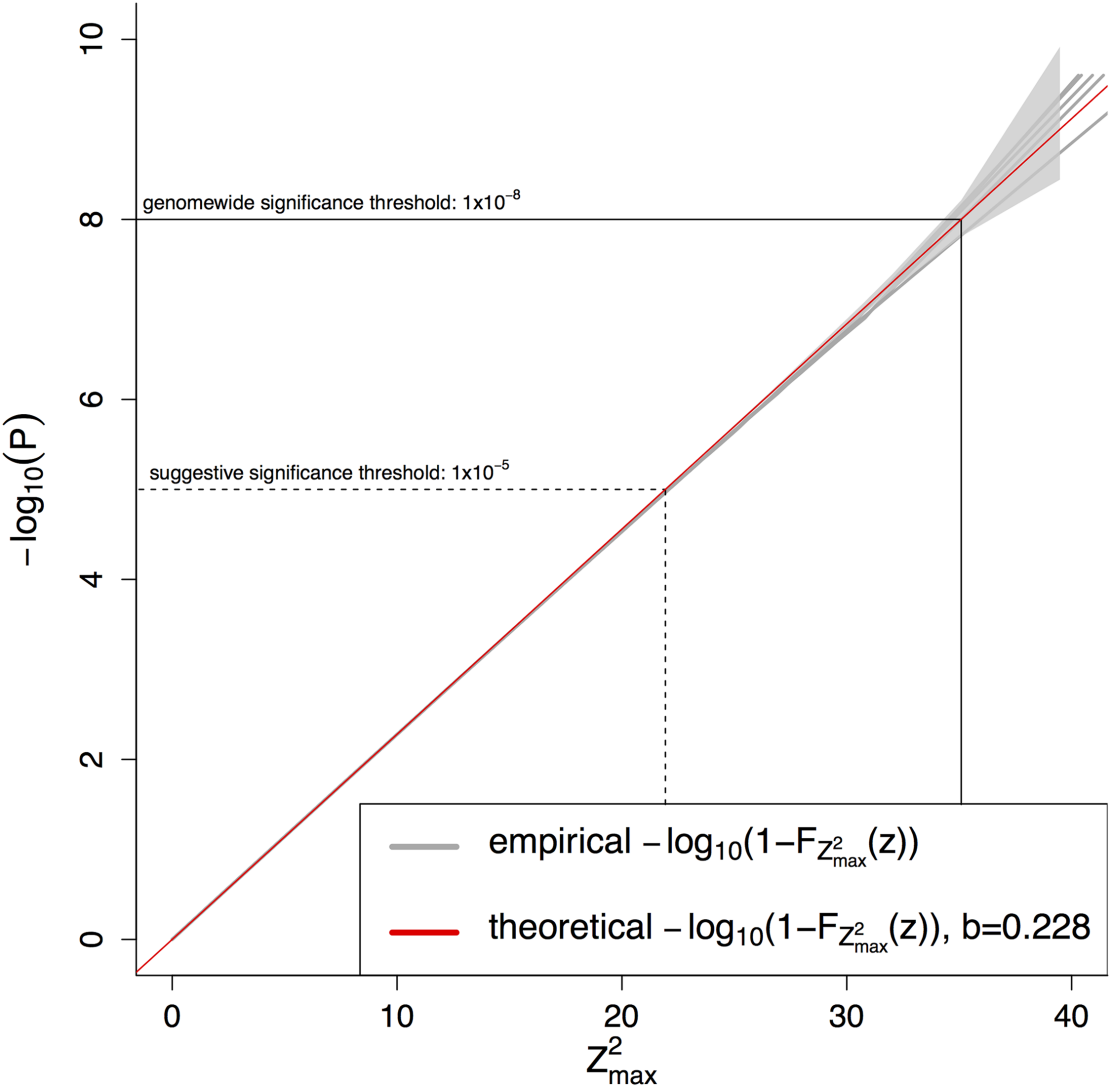
Five empirical evaluations of the −log_10_(*P*)-distribution of the $Z_{\text{max}}^{2}$ statistic, each obtained by simulating 2 × 10^9^ replicates. The theoretical distribution was obtained by fitting a straight line. The grey shaded area reflects the 95% Clopper-Pearson confidence interval [7].

Plotting −log_10_(*P*_*emp*_) against $Z_{max}^{2}$ suggests that the relationship can be expressed by a straight line (Fig 1).

A general formula for the distribution and density function of the maximum of independent identically-distributed (iid) variables has been described in Chapter 2.11 of Ewens & Grant [8]. Let *X*_1_, *X*_2_, …, *X*_*k*_ be continuous iid variables and *X*_max_ = max(*X*_1_, *X*_2_, …, *X*_*k*_) their maximum, then the cumulative distribution function of *X*_max_ can be written as follows:

$$\begin{array}{ccc} P(X_{\text{max}}\leq x) & = & P\left(X_{1}\leq x\cap X_{2}\leq x\cap \cdots \cap X_{k}\leq x\right)={\left\{P(X\leq x)\right\}}^{k}\\ & = & F_{X_{\text{max}}}\left(x\right)={\left\{F_{X}\left(x\right)\right\}}^{k} \end{array}$$ (5)

Formula (5) cannot be applied directly to our situation, since we do not have four independent variables. However, we can divide them into two independent blocks of iid $\chi_{1}^{2}$-distributed variables $Z_{1}^{2}⊥Z_{2}^{2}$ and $Z_{\text{12,agonistic}}^{2}⊥Z_{\text{12,antagonistic}}^{2}$. We let $F_{\chi_{1}^{2}}\left(z\right)$ be the distribution function of each variable $Z_{1}^{2},Z_{2}^{2},Z_{\text{12,agonistic}}^{2},Z_{\text{12,antagonistic}}^{2}$ and let $F_{Z_{\text{max}}^{2*}}\left(z\right)$ denote the distribution function of $Z_{\text{max}}^{2*}=\text{max}\left(Z_{1}^{2},Z_{2}^{2}\right)$ or $Z_{\text{max}}^{2*}=\text{max}\left(Z_{\text{12,agonistic}}^{2},Z_{\text{12,antagonistic}}^{2}\right)$, then

$$\begin{array}{c} F_{Z_{\text{max}}^{2*}}\left(z\right)={\left\{F_{\chi_{1}^{2}}\left(z\right)\right\}}^{2} \end{array}$$ (6)

Furthermore it is known that the sum of two iid $\chi_{1}^{2}$-distributed variables is $\chi_{2}^{2}$-distributed with the cumulative distribution function $F_{\chi_{2}^{2}}\left(z\right)$. Since we have only two independent random variables $Z_{1}^{2}$ and $Z_{2}^{2}$, we may postulate the following boundaries for $F_{Z_{\text{max}}^{2}}\left(z\right)$:

$$\begin{array}{c} F_{Z_{\text{max}}^{2*}}\left(z\right)\geq F_{Z_{\text{max}}^{2}}\left(z\right)\geq F_{\chi_{2}^{2}}\left(z\right) \end{array}$$ (7)

To prove that *F*_*Z*_*A*__(*z*) ≥ *F*_*Z*_*B*__(*z*) for two test statistics *Z*_*A*_ and *Z*_*B*_, we have to show that *Z*_*A*_ ≤ *Z*_*B*_ for every scenario, i.e., for every set of $Z_{1}^{2}$ and $Z_{2}^{2}$. It can be seen that $\text{max}\left(Z_{1}^{2},Z_{2}^{2}\right)\leq Z_{1}^{2}+Z_{2}^{2}$ and thus $F_{Z_{\text{max}}^{2*}}\left(z\right)\geq F_{\chi_{2}^{2}}\left(z\right)$. Furthermore, it is obvious that $\text{max}\left(Z_{1}^{2},Z_{2}^{2}\right)\leq \text{max}(Z_{1}^{2},Z_{2}^{2},\frac{{\left(Z_{1}+Z_{2}\right)}^{2}}{2},\frac{{\left(Z_{1}-Z_{2}\right)}^{2}}{2})$ and therefore $F_{Z_{\text{max}}^{2*}}\left(z\right)\geq F_{Z_{\text{max}}^{2}}\left(z\right)$. Finally, we prove that $F_{Z_{\text{max}}^{2}}\left(z\right)\geq F_{\chi_{2}^{2}}\left(z\right)$ by showing that $\text{max}(Z_{1}^{2},Z_{2}^{2},\frac{{\left(Z_{1}+Z_{2}\right)}^{2}}{2},\frac{{\left(Z_{1}-Z_{2}\right)}^{2}}{2})\leq Z_{1}^{2}+Z_{2}^{2}$. Since obviously $Z_{1}^{2}\leq Z_{1}^{2}+Z_{2}^{2}$ and $Z_{2}^{2}\leq Z_{1}^{2}+Z_{2}^{2}$, it remains to be shown that $\frac{{\left(Z_{1}+Z_{2}\right)}^{2}}{2}\leq Z_{1}^{2}+Z_{2}^{2}$ and $\frac{{\left(Z_{1}-Z_{2}\right)}^{2}}{2}\leq Z_{1}^{2}+Z_{2}^{2}$ (see S2 Appendix).

**Fig 2:**
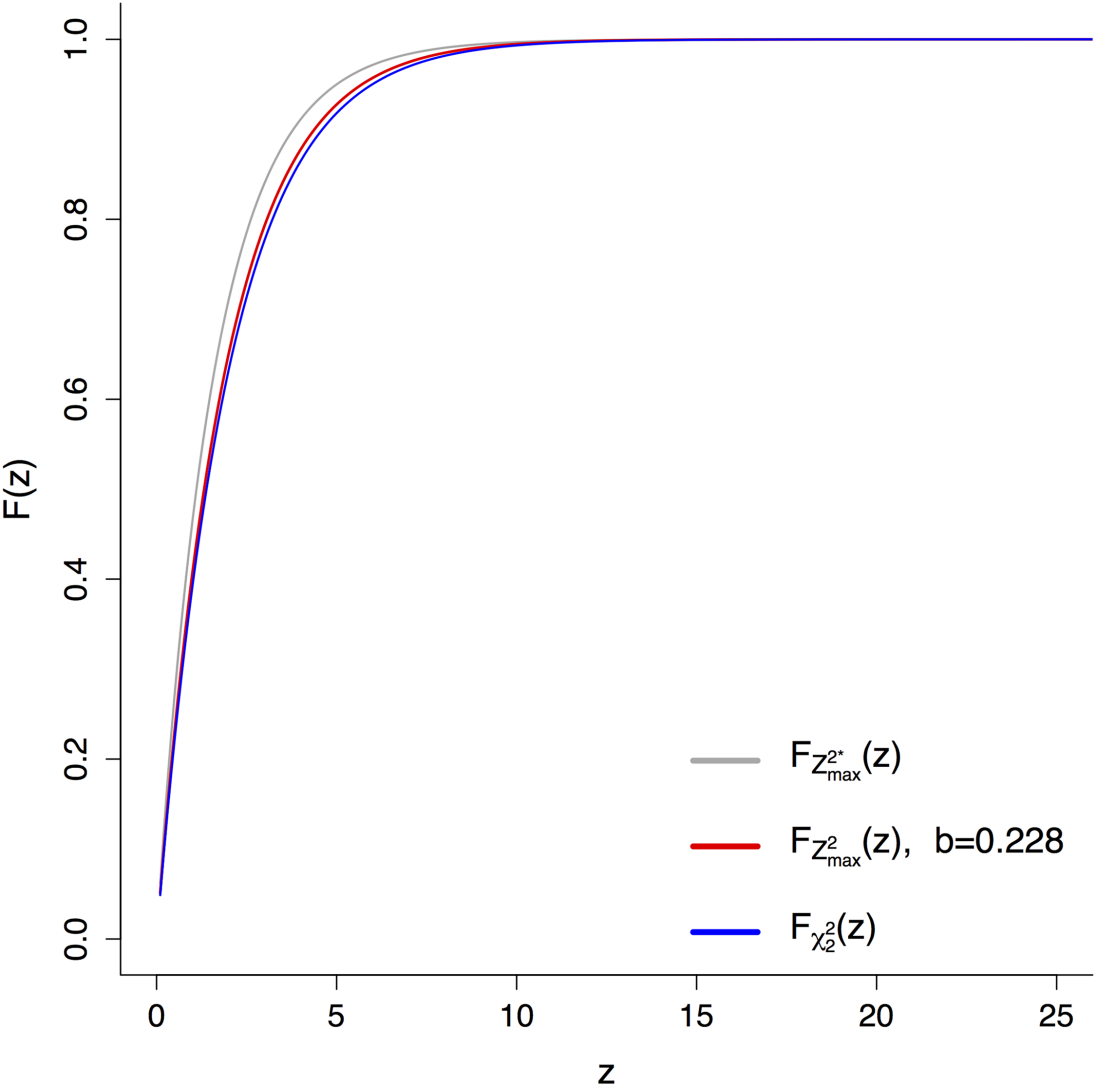
Comparison of $F_{Z_{\text{max}}^{2*}}\left(z\right)$, $F_{Z_{\text{max}}^{2}}\left(z\right)$ and $F_{\chi_{2}^{2}}\left(z\right)$.

This concludes the proof of Eq (7). Therefore, with Formula (7) we have established explicit boundaries for $F_{Z_{\text{max}}^{2}}\left(z\right)$, which are visualized in Fig 2.

It is important that $F_{Z_{\text{max}}^{2}}\left(z\right)$ is exponentially distributed. To derive that, note that $F_{\chi_{2}^{2}}\left(z\right)$ can be expressed in terms of an exponential distribution *F*_*λ*_(*z*) with scale parameter $\lambda =\frac{1}{2}$

$$\begin{array}{c} F_{\lambda }\left(z\right)=1-e^{-\lambda \cdot z} \end{array}$$ (8)

and *F*_*λ*_(*z*) is connected to *z* by a log-linear relation

$$\begin{array}{c} F_{\lambda }\left(z\right)=1-e^{-\lambda \cdot z}⟺-\log\left(1-F_{\lambda }\left(z\right)\right)=\lambda \cdot z \end{array}$$ (9)

Given the fact that the relationship of −log_10_(*P*) and $Z_{\text{max}}^{2}$ under H_0_ is a straight line (Fig 1), the cumulative distribution function of $Z_{\text{max}}^{2}$ is

$$\begin{array}{ccc} -\log_{10}\left(P\right) & = & b\cdot z\\ P & = & 10^{-b\cdot z}\\ F_{Z_{\text{max}}^{2}}\left(z\right)=1-P & = & 1-10^{-b\cdot z} \end{array}$$ (10)

Using the relationship 10^*x*^ = *e*^log(10)⋅*x*^, we can write $F_{Z_{\text{max}}^{2}}\left(z\right)$ as an exponential distribution

$$\begin{array}{ccc} F_{Z_{\text{max}}^{2}}\left(z\right) & = & 1-10^{-b\cdot z}\\ & = & 1-e^{-\log(10)\cdot b\cdot z}\\ & = & 1-e^{-\lambda z}\;\text{with}\;\lambda =\log\left(10\right)\cdot b \end{array}$$ (11)

In conclusion, from the empirically derived linear relation between the log_10_-transformed P-value and the test statistic it follows that $Z_{\text{max}}^{2}$ is exponentially distributed.

In order to determine the theoretical distribution, we searched for the optimal slope parameter *b*. To this end, we conducted two simulations of 100 empirical $Z_{\text{max}}^{2}$ distributions with R = 1,000,000,000 replicates and 5 empirical $Z_{\text{max}}^{2}$ distributions with R = 2,000,000,000 replicates, respectively. We estimated the slope parameter by means of linear regression and found a consistent estimate of *b* ≈ 0.228 (Table 1).

**Table 1:** Distribution of the slope parameter *b* of simulated $Z_{\text{max}}^{2}$ distributions by different simulation settings. sim. = simulations, repl. = replicates.

Setting	Min	Q1	Median	Q3	Max	Mean	Std Dev
100 sim.with 1 × 10^9^ repl.	0.22786	0.22795	0.22797	0.2280	0.22809	0.22797	3.88 ⋅ 10^−5^
5 sim. with 2 × 10^9^ repl.	0.22796	0.22797	0.22798	0.22798	0.22799	0.22798	1.08 ⋅ 10^−5^

With Eqs (10) and (11) we can give a formula for the cumulative distribution function of the original (not squared) *Z*_max_ statistic:

$$\begin{array}{ccc} F_{Z_{\text{max}}}\left(z\right) & = & 1-10^{-b\cdot z^{2}}\\ & = & 1-e^{-\log\left(10\right)\cdot b\cdot z^{2}},\;z\geq 0 \end{array}$$ (12)

Formula (12) represents the cumulative distribution function of the original *Z*_max_ statistic and we compare it with its simulated values from the previous study. We find theoretical thresholds for suggestive (10^−5^) and genomewide (10^−8^) significance of *Z*_max_ = 4.68 and *Z*_max_ = 5.92, respectively (S1 Fig). These thresholds correspond well to the values of 4.7 and 6 derived by our previous simulation study (see Methods section in Baurecht et al. [6]).

## Results

We compared the power and type 1 error (see S3 Appendix) of the CCMA method with the Subset-Based Meta-Analysis [5] implemented in the R-package ASSET [9] by simulations. To this end, we generated a fixed population of n = 20,000 individuals with respective genotypes according to the specified minor allele frequency (MAF) for a single SNV in exact Hardy-Weinberg Equilibrium. Then, we drew n = 8,000 individuals and simulated their phenotypes by applying a multinomial model with baseline risks for two diseases of 0.1 and 0.05 (e.g. AD and psoriasis), mimicking the respective prevalence using a previously described algorithm [10]. For simplicity the controls were distributed equally between both case sets. We varied the minor allele frequencies (MAF) ∈ (0.1, 0.2, 0.3) and the odds ratios (OR) ∈ (1.15, 1.2, 1.3). Power was estimated for levels of *α* = 0.001 and *α* = 10^−5^ with R = 1,000 replicates to detect (a) disease specific, (b) agonistic and (c) antagonistic effects.

In the simulation-based power analysis we found that the CCMA method is only marginally less powerful for detecting disease specific, agonistic and antagonistic effects than the ASSET method (S2, S3, S4 Figs, Table 2). However, CCMA provides better control over the type 1 error rate (see S1 Table and S5 Fig). These results demonstrate the trade off between power and controlling type 1 error. If we would use e.g. the inflated ASSET threshold of 0.01205 for CCMA (S1 Table: OR = 1.3, MAF = 0.2, *α* = 0.01), then ASSET and CCMA exhibit almost identical power (disease-specific: Power_ASSET_ = 0.830, Power_CCMA_ = 0.839; agonistic: Power_ASSET_ = 0.976, Power_CCMA_ = 0.974; antagonistic: Power_ASSET_ = 0.952, Power_CCMA_ = 0.955). We obtained comparable results by setting equal baseline risks for both diseases (data not shown).

**Table 2:** Power comparison of the CCMA and Subset-Based Meta-Analysis (ASSET) for detection of true associations at a significance level of α = 0.001 and α = 10^−5^. For each power estimate, we ran R = 1,000 simulations with n = 8,000 individuals for various MAF and OR values and assigned the disease status by a multinomial model.

MAF	OR	disease-specific effect		agonistic effect		antagonistic effect	
		ASSET	CCMA	ASSET	CCMA	ASSET	CCMA
*α* = 0.001							
0.1	1.15	0.0320	0.0270	0.0600	0.0520	0.0430	0.0360
	1.2	0.0900	0.0860	0.1620	0.1400	0.1140	0.1060
	1.3	0.2760	0.2660	0.5780	0.5420	0.4470	0.4330
0.2	1.15	0.0780	0.0690	0.1820	0.1700	0.1340	0.1300
	1.2	0.1760	0.1730	0.4430	0.4160	0.3450	0.3270
	1.3	0.6200	0.6070	0.9050	0.8920	0.8320	0.8200
0.3	1.15	0.1100	0.1090	0.2460	0.2240	0.2130	0.2000
	1.2	0.2950	0.2830	0.6130	0.5830	0.5330	0.5060
	1.3	0.8170	0.8150	0.9760	0.9670	0.9430	0.9360
*α* = 10^−5^							
0.1	1.15	0.0010	0.0010	0.0030	0.0020	0.0010	0.0020
	1.2	0.0080	0.0100	0.0220	0.0220	0.0140	0.0110
	1.3	0.0540	0.0540	0.1980	0.1880	0.0940	0.0910
0.2	1.15	0.0080	0.0090	0.0190	0.0190	0.0070	0.0070
	1.2	0.0240	0.0260	0.1010	0.0900	0.0630	0.0580
	1.3	0.2320	0.2280	0.5800	0.5540	0.4490	0.4210
0.3	1.15	0.0130	0.0100	0.0300	0.0260	0.0230	0.0240
	1.2	0.0560	0.0540	0.2090	0.1940	0.1380	0.1290
	1.3	0.4160	0.4190	0.8000	0.7830	0.6960	0.6790

A minor modification of the CCMA test statistic allows taking study size into account by using weights *w*_1_ and *w*_2_ (see S4 Appendix), which improves power for detecting either agonistic or antagonistic effects, depending on the specification of the transformation matrix (S2 Table).

If we distribute the controls in proportion to the case sets, which is a reasonable scenario in practice, the power of both methods is mostly increased. Of note, for disease specific and antagonistic effects and *α* = 10^−5^ the power of CCMA and its modified version is in most cases higher than the power of ASSET (S3 Table).

## Discussion

We have previously shown that the CCMA method is an appealing approach to screen for shared and disease-specific loci as well as to leverage additional cross-phenotype association information using available GWAS data [6]. We have now determined the null distribution for the CCMA test statistic, which corresponds to an exponential distribution, and we show that CCMA demonstrates comparable power for detecting disease-specific, agonistic and antagonistic loci to the frequently used Subset-Based Meta-Analysis [5] (ASSET) approach, while better controlling the type I error. The CCMA method, which is calculated in a straightforward way, allows us to infer the mode of pleiotropy directly by looking at which of the four constituent statistics *T*_1_, *T*_2_, *T*_12,agonistic_ or *T*_12,antagonistic_ yields the maximum. Finally, the CCMA method can also be applied to other genome-wide molecular data (e.g. gene expression, epigenomics, metabolomics) as well as to other research questions such as those encountered in environmental epidemiology. Here, the influence of environmental exposures or lifestyle factors on two different traits of interest can be analyzed with regard to their concordant or contrasting effects.

In subgroup meta-analysis similar questions are addressed by e.g. comparing group A vs. group B using a Z-test ${\text{Z}}_{\text{Diff}}=\left({\text{eff}}_{\text{A}}-{\text{eff}}_{\text{B}}\right)/\sqrt{\text{Var}\left({\text{eff}}_{\text{A}}\right)+\text{Var}\left({\text{eff}}_{\text{B}}\right)}$ [11]. This Z-test allows only to contrast two effects, but neither to consider disease-specific, agonistic and antagonistic effects simultaneously nor to distinguish between them. A canonical method to approach such questions would be a multinomial regression model followed by Wald tests for testing effect contrasts [12]. Although the multinomial regression model allows to incorporate covariates, it is not applicable if only summary statistics are available and it requires by far more computing time if applied on a genome-wide level.

In conclusion, the proposed CCMA method has some attractive properties for investigating the effect of exposure variables on two different traits. The simply constructed test statistic follows an exponential distribution under the null hypothesis, which allows a fast and easy implementation as well as a direct deduction of the mode of pleiotropy. The method can be conveniently applied to similar questions in other domains and can also exploit summary statistics from two single studies.

## Supporting Information

S1 Fig Empirical and theoretical −log_10_(P)-distribution of Z_max_ with parameter b = 0.228. Dotted and solid grey lines indicate the thresholds of suggestive (*Z*_max_ = 4.68) and genomewide significance (*Z*_max_ = 5.92).
(TIF)

S2 Fig Simulation-based power comparison of CCMA and Subset-Based Meta-Analysis (ASSET) for detecting a disease-specific effect. For each power estimate, we ran R = 1,000 simulations with n = 8,000 individuals for various MAF and OR values and assigned the disease status by a multinomial model. A significance threshold of *α* = 0.001 and *α* = 10^−5^ was applied.
(PDF)

S3 Fig Simulation-based power comparison of CCMA and Subset-Based Meta-Analysis (ASSET) for detecting an agonistic effect. For each power estimate, we ran R = 1,000 simulations with n = 8,000 individuals for various MAF and OR values and assigned the disease status by a multinomial model. A significance threshold of *α* = 0.001 and *α* = 10^−5^ was applied.
(PDF)

S4 Fig Simulation-based power comparison of CCMA and Subset-Based Meta-Analysis (ASSET) for detecting an antagonistic effect. For each power estimate, we ran R = 1,000 simulations with n = 8,000 individuals for various MAF and OR values and assigned the disease status by a multinomial model. A significance threshold of *α* = 0.001 and *α* = 10^−5^ was applied.
(PDF)

S5 Fig Simulation-based type 1 error comparison of CCMA, wCCMA and the Subset-Based Meta-Analysis (ASSET) under H_0_. We ran R = 100,000 simulations with n = 8,000 individuals for various MAF values under H_0_. Several significance thresholds were considered for comparison *α* = (0.001, 0.005, 0.01, 0.05).
(PDF)

S1 Table Type 1 error comparison of CCMA, wCCMA and the Subset-Based Meta-Analysis (ASSET) under H_0_. We ran R = 100,000 simulations with n = 8,000 individuals for various MAF under H_0_. Several significance thresholds were considered for comparison *α* = (0.001, 0.005, 0.01, 0.05).
(PDF)

S2 Table Power comparison of the CCMA, wCCMA and Subset-Based Meta-Analysis (ASSET) for detection of true associations at a significance level of α = 0.001 and α = 10^−5^. For each power estimate, we ran R = 1,000 simulations with n = 8,000 individuals for various MAF and OR values and assigned the disease status by a multinomial model and distributed controls equally to both case sets.
(PDF)

S3 Table Power comparison of the CCMA, wCCMA and Subset-Based Meta-Analysis (ASSET) for detection of true associations at a significance level of α = 0.001 and α = 10^−5^. For each power estimate, we ran R = 1,000 simulations with n = 8,000 individuals for various MAF and OR values and assigned the disease status by a multinomial model and distributed controls proportionally to the case sets.
(PDF)

S1 Appendix Proof of Independence between *Z*_12,agonistic_ and *Z*_12,antagonistic_. (PDF)

S2 AppendixProof that $\frac{{\left(Z_{1}+Z_{2}\right)}^{2}}{2}\leq Z_{1}^{2}+Z_{2}^{2}$ and $\frac{{\left(Z_{1}-Z_{2}\right)}^{2}}{2}\leq Z_{1}^{2}+Z_{2}^{2}$.(PDF)

S3 Appendix Comparison of the Type 1 Error. (PDF)

S4 Appendix Weighted CCMA Test Statistic (wCCMA). (PDF)

## References

[1] Sivakumaran S, Agakov F, Theodoratou E, Prendergast JG, Zgaga L, Manolio T, et al Abundant pleiotropy in human complex diseases and traits. Am J Hum Genet. 2011;89(5):607–618. 10.1016/j.ajhg.2011.10.004 22077970 PMC3213397

[2] Arnold M, Hartsperger ML, Baurecht H, Rodríguez E, Wachinger B, Franke A, et al Network-based SNP meta-analysis identifies joint and disjoint genetic features across common human diseases. BMC Genomics. 2012;13:490 10.1186/1471-2164-13-490 22988944 PMC3782362

[3] Welter D, MacArthur J, Morales J, Burdett T, Hall P, Junkins H, et al The NHGRI GWAS Catalog, a curated resource of SNP-trait associations. Nucleic Acids Res. 2014;42(Database issue):D1001–D1006. 10.1093/nar/gkt1229 24316577 PMC3965119

[4] Ellinghaus D, Ellinghaus E, Nair RP, Stuart PE, Esko T, Metspalu A, et al Combined analysis of genome-wide association studies for Crohn disease and psoriasis identifies seven shared susceptibility loci. Am J Hum Genet. 2012;90(4):636–647. 10.1016/j.ajhg.2012.02.020 22482804 PMC3322238

[5] Bhattacharjee S, Rajaraman P, Jacobs KB, Wheeler WA, Melin BS, Hartge P, et al A subset-based approach improves power and interpretation for the combined analysis of genetic association studies of heterogeneous traits. Am J Hum Genet. 2012;90(5):821–835. 10.1016/j.ajhg.2012.03.015 22560090 PMC3376551

[6] Baurecht H, Hotze M, Brand S, Büning C, Cormican P, Corvin A, et al Genome-wide Comparative Analysis of Atopic Dermatitis and Psoriasis Gives Insight into Opposing Genetic Mechanisms. Am J Hum Genet. 2015;96(1):104–120. 10.1016/j.ajhg.2014.12.004 25574825 PMC4289690

[7] Clopper C, Pearson ES. The use of confidence or fiducial limits illustrated in the case of the binomial. Biometrika. 1934;26:404–13. 10.1093/biomet/26.4.404

[8] Ewens W, Grant G. Statisical Methods in Bioinformatics: An Introduction 2nd ed Gail M, Krickeberg K, Samet J, editors. Statistics for Biology and Health. New York: Springer; 2005.

[9] Bhattacharjee S, Chatterjee N, Wheeler W. ASSET: An R package for subset-based association analysis of heterogeneous traits and subtypes; 2013.

[10] Smart F. Simulating Multinomial logit in Stata—Updated; 2012. Available from: http://www.econometricsbysimulation.com/2012/07/simulating-multinomial-logit-in-stata.html

[11] Borenstein M, Hedges LV, Higgins JPT, Rothstein HR. Subgroup Analysis In: Introduction to Meta-Analysis. West Sussex: Wiley & Sons; 2009 p. 156–57.

[12] Fahrmeir L, Tutz G. Models for Multicategorical Responses: Multivariate Extensions of Generalized Linear Models In: Multivariate Statistical Modelling Based on Generalized Linear Models. 2nd ed Springer; 2001 p. 107.

