# Supplementary figures and images for: Compare and Contrast Meta Analysis (CCMA): A Method for Identification of Pleiotropic Loci in Genome-Wide Association Studies
Source: PLoS One. 2016 May 5;11(5):e0154872. doi: 10.1371/journal.pone.0154872 (PMC4858294; doi:10.1371/journal.pone.0154872)

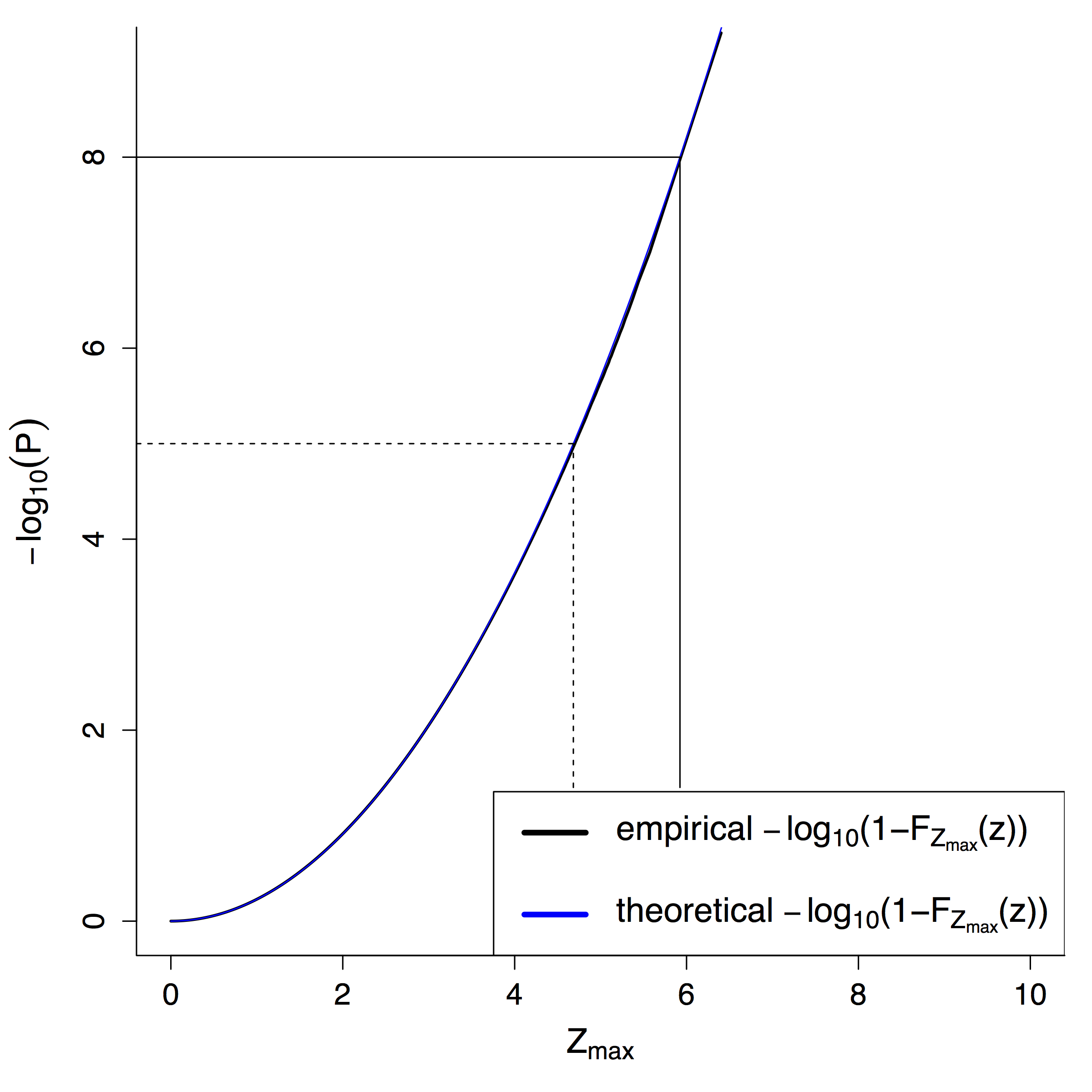

Supplement: S1 Fig — Dotted and solid grey lines indicate the thresholds of suggestive (Zmax = 4.68) and genomewide significance (Zmax = 5.92). (TIF) [file pone.0154872.s001.tif]

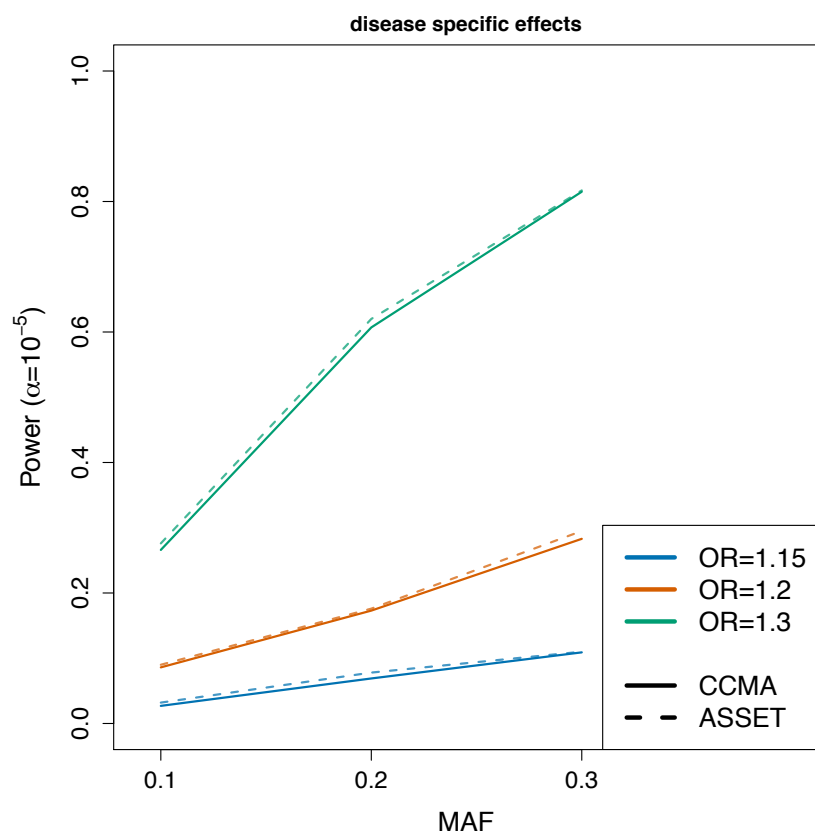

(a)  $\alpha = 0.001$

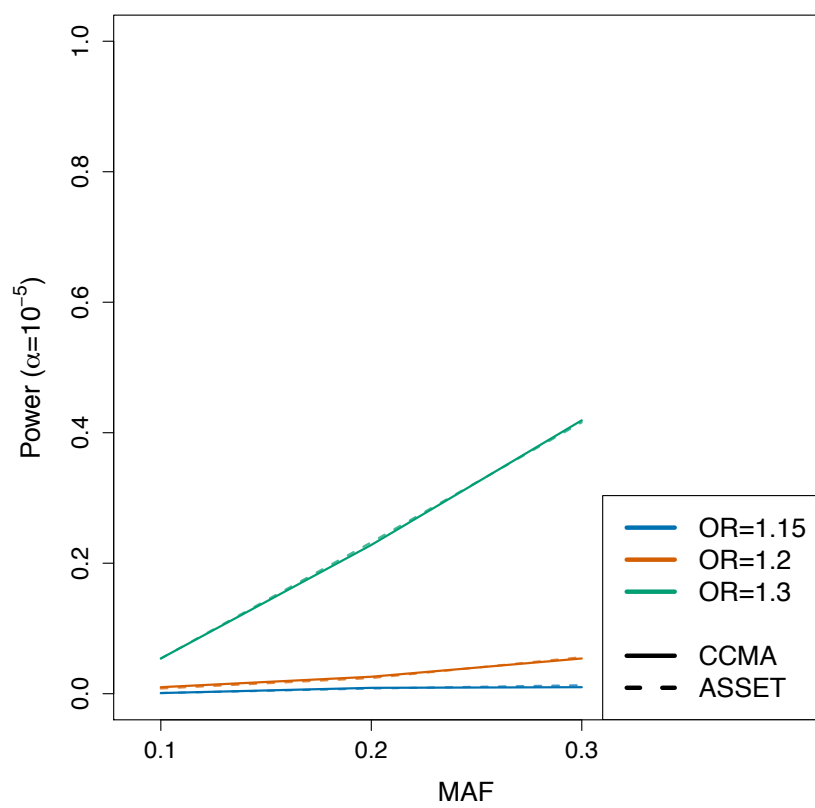

(b)  $\alpha = 10^{-5}$

Supplement: S2 Fig — For each power estimate, we ran R = 1,000 simulations with n = 8,000 individuals for various MAF and OR values and assigned the disease status by a multinomial model. A significance threshold of α = 0.001 and α = 10−5 was applied. (PDF) [file pone.0154872.s002.pdf]

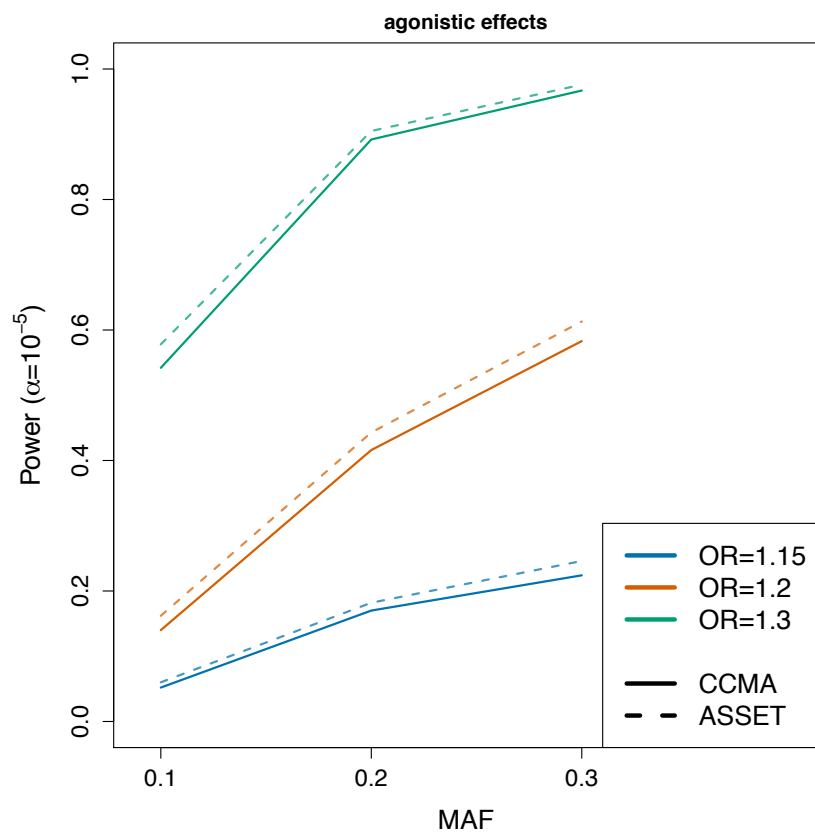

(a)  $\alpha = 0.001$

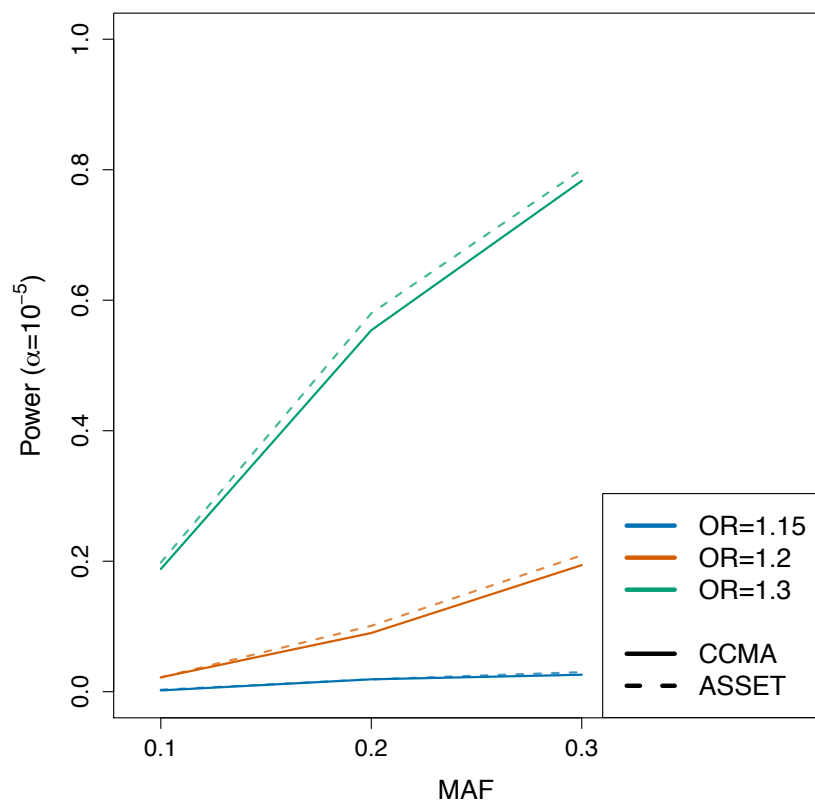

(b)  $\alpha = 10^{-5}$

Supplement: S3 Fig — For each power estimate, we ran R = 1,000 simulations with n = 8,000 individuals for various MAF and OR values and assigned the disease status by a multinomial model. A significance threshold of α = 0.001 and α = 10−5 was applied. (PDF) [file pone.0154872.s003.pdf]

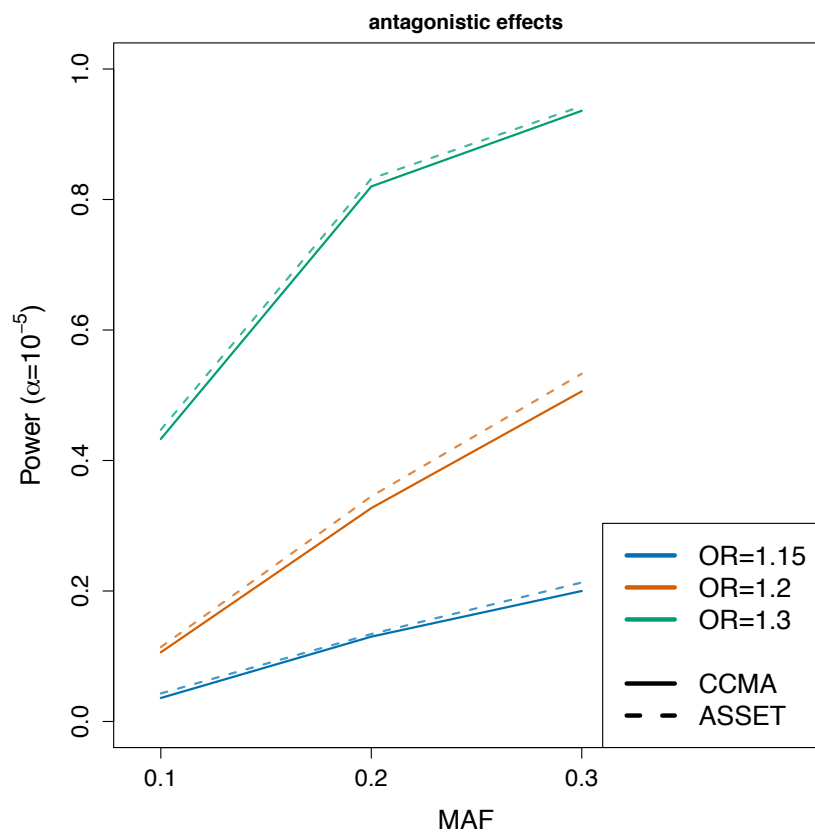

(a)  $\alpha = 0.001$

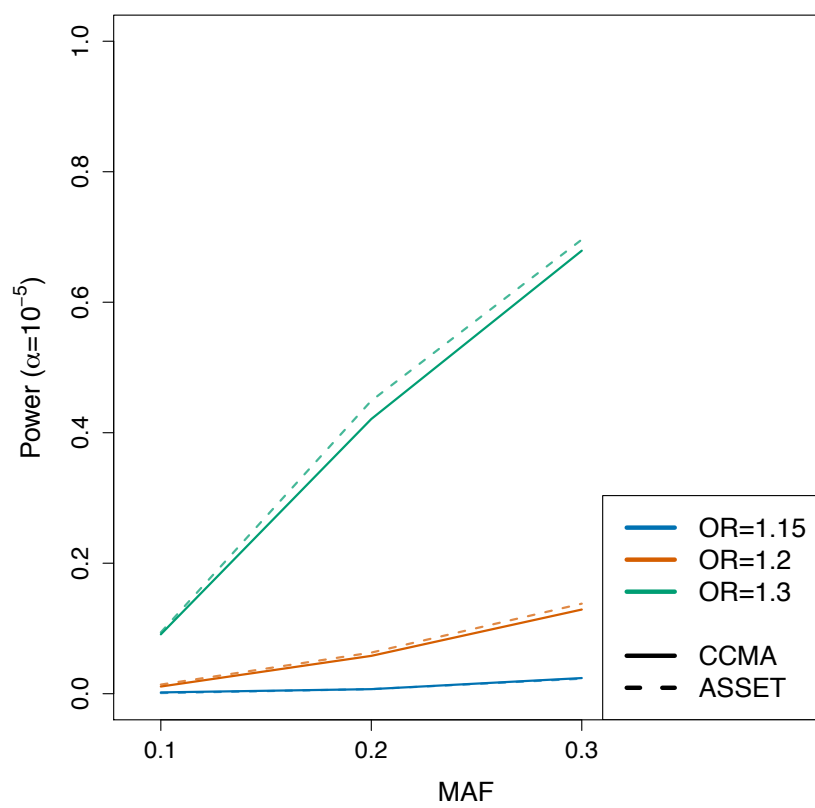

(b)  $\alpha = 10^{-5}$

Supplement: S4 Fig — For each power estimate, we ran R = 1,000 simulations with n = 8,000 individuals for various MAF and OR values and assigned the disease status by a multinomial model. A significance threshold of α = 0.001 and α = 10−5 was applied. (PDF) [file pone.0154872.s004.pdf]

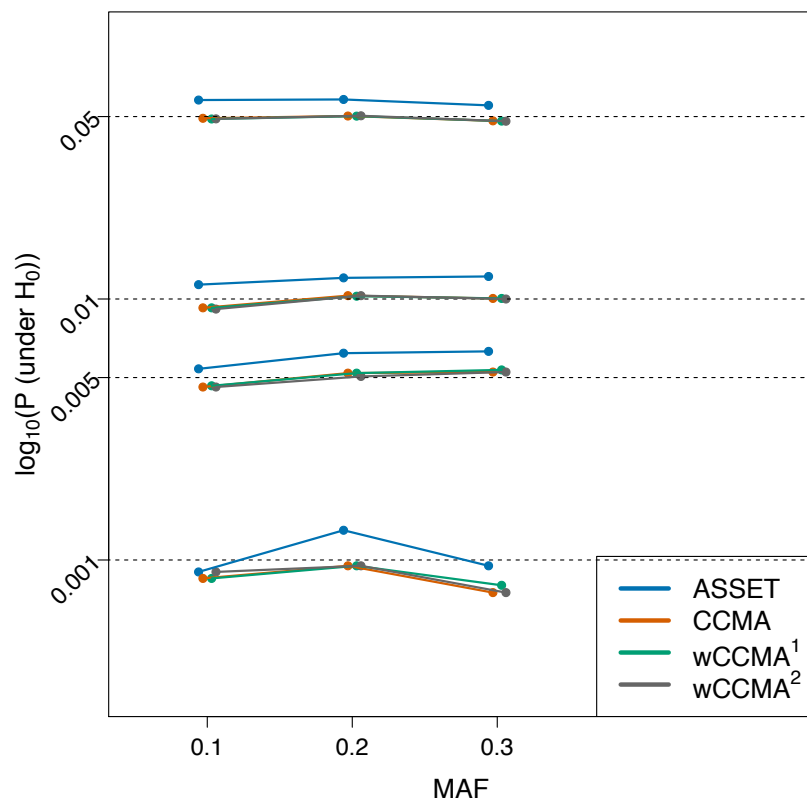

(a) equally distributed controls

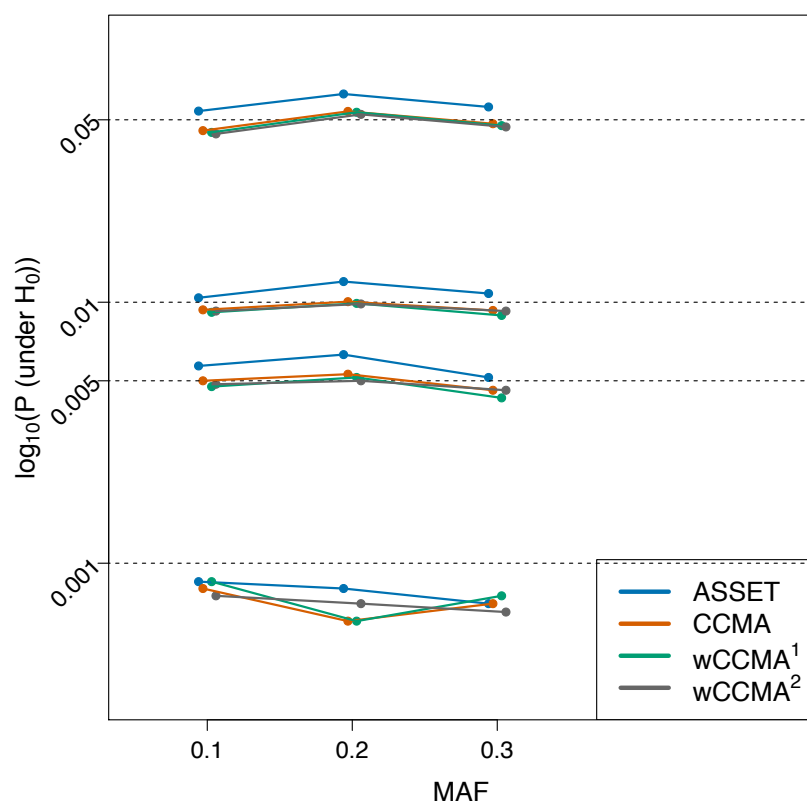

(b) proportionally distributed controls

Supplement: S5 Fig — We ran R = 100,000 simulations with n = 8,000 individuals for various MAF values under H0. Several significance thresholds were considered for comparison α = (0.001, 0.005, 0.01, 0.05). (PDF) [file pone.0154872.s005.pdf]
