# Supplementary material for: Compare and Contrast Meta Analysis (CCMA): A Method for Identification of Pleiotropic Loci in Genome-Wide Association Studies
Source: PLoS One. 2016 May 5;11(5):e0154872. doi: 10.1371/journal.pone.0154872 (PMC4858294; doi:10.1371/journal.pone.0154872)
Supplement: S1 Table — We ran R = 100,000 simulations with n = 8,000 individuals for various MAF under H0. Several significance thresholds were considered for comparison α = (0.001, 0.005, 0.01, 0.05). (PDF) [file pone.0154872.s006.pdf]

		controls equally distributed				controls proportionally distributed			
MAF	α	ASSET	CCMA	wCCMA[1]	wCCMA[2]	ASSET	CCMA	wCCMA[1]	wCCMA[2]
0.1	0.001	0.00090	0.00085	0.00085	0.00090	0.00085	0.00080	0.00085	0.00075
	0.005	0.00540	0.00465	0.00465	0.00460	0.00570	0.00500	0.00475	0.00485
	0.01	0.01135	0.00925	0.00925	0.00915	0.01040	0.00935	0.00915	0.00925
	0.05	0.05785	0.04925	0.04895	0.04900	0.05400	0.04545	0.04475	0.04415
0.2	0.001	0.00130	0.00095	0.00095	0.00195	0.00080	0.00060	0.00060	0.00070
	0.005	0.00620	0.00520	0.00520	0.00505	0.00630	0.00530	0.00515	0.00500
	0.01	0.01205	0.01030	0.01025	0.01030	0.01200	0.01005	0.00990	0.00985
	0.05	0.05815	0.05025	0.05020	0.05035	0.06280	0.05385	0.05345	0.05245
0.3	0.001	0.00095	0.00075	0.00080	0.00075	0.00070	0.00070	0.00075	0.00065
	0.005	0.00630	0.00525	0.00535	0.00525	0.00515	0.00460	0.00430	0.00460
	0.01	0.01220	0.01005	0.01005	0.01000	0.01080	0.00930	0.00890	0.00925
	0.05	0.05520	0.04815	0.04805	0.04800	0.05600	0.04830	0.04750	0.04695

[1]wCCMA using transformation matrix $\mathbf{A}_{(1)}$

[2]wCCMA using transformation matrix $\mathbf{A}_{(2)}$
