# Supplementary material for: Compare and Contrast Meta Analysis (CCMA): A Method for Identification of Pleiotropic Loci in Genome-Wide Association Studies
Source: PLoS One. 2016 May 5;11(5):e0154872. doi: 10.1371/journal.pone.0154872 (PMC4858294; doi:10.1371/journal.pone.0154872)
Supplement: S2 Table — For each power estimate, we ran R = 1,000 simulations with n = 8,000 individuals for various MAF and OR values and assigned the disease status by a multinomial model and distributed controls equally to both case sets. (PDF) [file pone.0154872.s007.pdf]

MAF	OR	disease-specific effect				agonistic effect				antagonistic effect			
		ASSET	CCMA	wCCMA[1]	wCCMA[2]	ASSET	CCMA	wCCMA[1]	wCCMA[2]	ASSET	CCMA	wCCMA[1]	wCCMA[2]
$\alpha = 0.001$													
0.1	1.15	0.0320	0.0270	0.0270	0.0270	0.0600	0.0520	0.0530	0.0510	0.0430	0.0360	0.0350	0.0360
	1.2	0.0900	0.0860	0.0870	0.0860	0.1620	0.1400	0.1420	0.1410	0.1140	0.1060	0.1050	0.1100
	1.3	0.2760	0.2660	0.2670	0.2650	0.5780	0.5420	0.5430	0.5400	0.4470	0.4330	0.4310	0.4350
0.2	1.15	0.0780	0.0690	0.0690	0.0690	0.1820	0.1700	0.1700	0.1710	0.1340	0.1300	0.1280	0.1300
	1.2	0.1760	0.1730	0.1720	0.1720	0.4430	0.4160	0.4190	0.4150	0.3450	0.3270	0.3250	0.3330
	1.3	0.6200	0.6070	0.6070	0.6090	0.9050	0.8920	0.8930	0.8900	0.8320	0.8200	0.8170	0.8220
0.3	1.15	0.1100	0.1090	0.1080	0.1110	0.2460	0.2240	0.2240	0.2270	0.2130	0.2000	0.1950	0.1980
	1.2	0.2950	0.2830	0.2820	0.2830	0.6130	0.5830	0.5870	0.5790	0.5330	0.5060	0.5040	0.5140
	1.3	0.8170	0.8150	0.8150	0.8140	0.9760	0.9670	0.9680	0.9660	0.9430	0.9360	0.9340	0.9390
$\alpha = 10^{-5}$													
0.1	1.15	0.0010	0.0010	0.0010	0.0010	0.0030	0.0020	0.0020	0.0020	0.0010	0.0020	0.0020	0.0020
	1.2	0.0080	0.0100	0.0100	0.0100	0.0220	0.0220	0.0220	0.0220	0.0140	0.0110	0.0110	0.0120
	1.3	0.0540	0.0540	0.0530	0.0530	0.1980	0.1880	0.1860	0.1870	0.0940	0.0910	0.0900	0.0910
0.2	1.15	0.0080	0.0090	0.0090	0.0090	0.0190	0.0190	0.0180	0.0200	0.0070	0.0070	0.0070	0.0070
	1.2	0.0240	0.0260	0.0260	0.0260	0.1010	0.0900	0.0910	0.0910	0.0630	0.0580	0.0570	0.0590
	1.3	0.2320	0.2280	0.2280	0.2290	0.5800	0.5540	0.5590	0.5520	0.4490	0.4210	0.4120	0.4290
0.3	1.15	0.0130	0.0100	0.0100	0.0100	0.0300	0.0260	0.0270	0.0250	0.0230	0.0240	0.0240	0.0230
	1.2	0.0560	0.0540	0.0540	0.0540	0.2090	0.1940	0.1950	0.1930	0.1380	0.1290	0.1250	0.1340
	1.3	0.4160	0.4190	0.4190	0.4180	0.8000	0.7830	0.7820	0.7810	0.6960	0.6790	0.6690	0.6830

[1]wCCMA using transformation matrix $\mathbf{A}_{(1)}$

[2]wCCMA using transformation matrix $\mathbf{A}_{(2)}$
