# Supplementary material for: Compare and Contrast Meta Analysis (CCMA): A Method for Identification of Pleiotropic Loci in Genome-Wide Association Studies
Source: PLoS One. 2016 May 5;11(5):e0154872. doi: 10.1371/journal.pone.0154872 (PMC4858294; doi:10.1371/journal.pone.0154872)
Supplement: S1 Appendix — (PDF) [file pone.0154872.s009.pdf]

Appendix S1. Proof of Independence between $Z_{\mathbf{12,agonistic}}$ and $Z_{\mathbf{12,antagonistic}}$

To prove that $Z_{12,\text{agonistic}} \perp Z_{12,\text{antagonistic}}$, we show that $\mathbf{y} = (Z_{12,\text{agonistic}}, Z_{12,\text{antagonistic}})^T \sim N_2(\mu_{\mathbf{y}}, \mathbf{\Sigma_y})$ with the identity matrix $\mathbf{\Sigma_y} = \left(\begin{smallmatrix} 1 & 0 \\ 0 & 1 \end{smallmatrix}\right)$. To this end, we use the theorem (4.3) in Dickhaus [1] on page 50:

Theorem 1 Let $\mathbf{x} \sim N_p(\mu_{\mathbf{x}}, \mathbf{\Sigma_x})$ and $\mathbf{y} = \mathbf{Ax} + \mathbf{b}$, in which $\mathbf{A}$ is a $(q \times p)$-matrix with $\text{rank}(\mathbf{A}) = q \leq p$. Then $\mathbf{y} \sim N_q(\mu_{\mathbf{y}}, \mathbf{\Sigma_y})$ with $\mu_{\mathbf{y}} = \mathbf{A}\mu_{\mathbf{x}} + \mathbf{b}$ and $\mathbf{\Sigma_y} = \mathbf{A\Sigma_x A}^T$.

We let $\mathbf{x} = (Z_1, Z_2)^T$ be $N_2(\mu_{\mathbf{x}}, \mathbf{\Sigma_x})$ distributed with $\mu_{\mathbf{x}} = \left(\begin{smallmatrix} 0 \\ 0 \end{smallmatrix}\right)$ and $\mathbf{\Sigma_x} = \left(\begin{smallmatrix} 1 & 0 \\ 0 & 1 \end{smallmatrix}\right)$. Then, $\mathbf{y} = (Z_{12,\text{agonistic}}, Z_{12,\text{antagonistic}})^T = \mathbf{Ax} + \mathbf{b}$ with $\mathbf{A} = \frac{1}{\sqrt{2}} \left(\begin{smallmatrix} 1 & 1 \\ 1 & \text{-}1 \end{smallmatrix}\right)$ and $\mathbf{b} = \left(\begin{smallmatrix} 0 \\ 0 \end{smallmatrix}\right)$.

It can be seen that $E(\mathbf{y}) = \mu_{\mathbf{y}} = \mathbf{A}\mu_{\mathbf{x}} + \mathbf{b} = \left(\begin{smallmatrix} 0 \\ 0 \end{smallmatrix}\right)$ with variance

$$\begin{aligned}
\mathbf{\Sigma_y} &= \mathbf{A\Sigma_x A}^T \\
&= \frac{1}{\sqrt{2}} \begin{pmatrix} 1 & 1 \\ 1 & -1 \end{pmatrix} \begin{pmatrix} 1 & 0 \\ 0 & 1 \end{pmatrix} \begin{pmatrix} 1 & 1 \\ 1 & -1 \end{pmatrix} \frac{1}{\sqrt{2}} \\
&= \frac{1}{2} \begin{pmatrix} 1 & 1 \\ 1 & -1 \end{pmatrix} \begin{pmatrix} 1 & 1 \\ 1 & -1 \end{pmatrix} \\
&= \begin{pmatrix} 1 & 0 \\ 0 & 1 \end{pmatrix}.
\end{aligned}$$

Reference for Appendix S1

1. Dickhaus T. Simultaneous Statistical Inference: With Applications in the Life Sciences. vol. 1. Heidelberg - New York: Springer; 2014.
