# Supplementary material for: Compare and Contrast Meta Analysis (CCMA): A Method for Identification of Pleiotropic Loci in Genome-Wide Association Studies
Source: PLoS One. 2016 May 5;11(5):e0154872. doi: 10.1371/journal.pone.0154872 (PMC4858294; doi:10.1371/journal.pone.0154872)
Supplement: S2 Appendix — (PDF) [file pone.0154872.s010.pdf]

Appendix S2. Proof that $\frac{(Z_1+Z_2)^2}{2} \leq Z_1^2 + Z_2^2$ and $\frac{(Z_1-Z_2)^2}{2} \leq Z_1^2 + Z_2^2$

$$\begin{aligned}
\frac{(Z_1+Z_2)^2}{2} &\leq Z_1^2 + Z_2^2 \\
\frac{Z_1^2 + 2Z_1Z_2 + Z_2^2}{2} &\leq Z_1^2 + Z_2^2 \\
\frac{Z_1^2 + Z_2^2}{2} + Z_1Z_2 &\leq Z_1^2 + Z_2^2 \\
Z_1Z_2 &\leq \frac{Z_1^2 + Z_2^2}{2} \\
Z_1^2 - 2\,Z_1Z_2 + Z_2^2 &\geq 0 \\
(Z_1 - Z_2)^2 &\geq 0 \quad \text{(agonistic case)}
\end{aligned}$$

This obviously holds irrespective of the values of the test statistics Z_1^2 and Z_2^2. For the antagonistic case, we only have to change the sign in the left term, which results in

$$(Z_1 + Z_2)^2 \geq 0 \quad \text{(antagonistic case)}$$

which also holds for any values of Z_1^2 and Z_2^2.
