# Supplementary material for: Compare and Contrast Meta Analysis (CCMA): A Method for Identification of Pleiotropic Loci in Genome-Wide Association Studies
Source: PLoS One. 2016 May 5;11(5):e0154872. doi: 10.1371/journal.pone.0154872 (PMC4858294; doi:10.1371/journal.pone.0154872)
Supplement: S3 Appendix — (PDF) [file pone.0154872.s011.pdf]

Appendix S3. Comparison of the Type 1 Error

We also compared type 1 error between CCMA, wCCMA and ASSET under the null hypothesis H_0 of of no pleiotropy and no association between genetic markers and any disease. To this end we simulated 100,000 replicates with the settings mentioned above and (a) equally distributed and (b) proportionally distributed controls to both case sets.

Comparison of the type 1 error under H_0 showed that the CCMA and the wCCMA methods hold the significance levels at or slightly below the thresholds. In contrast, the Subset-based method (ASSET) shows inflated P-values compared to CCMA and wCCMA in all settings. (Table S1, Figure S5)
