# Supplementary material for: Compare and Contrast Meta Analysis (CCMA): A Method for Identification of Pleiotropic Loci in Genome-Wide Association Studies
Source: PLoS One. 2016 May 5;11(5):e0154872. doi: 10.1371/journal.pone.0154872 (PMC4858294; doi:10.1371/journal.pone.0154872)
Supplement: S4 Appendix — (PDF) [file pone.0154872.s012.pdf]

Appendix S4. Weighted CCMA Test Statistic (wCCMA)

The CCMA test statistic can be modified to incorporate weights taking into account the study size; this will be termed wCCMA. We let $w_k = \sqrt{\frac{N_k}{N}}$ be the weights for study k and $\mathbf{x} = (T_1, T_2)^T$. Then the transformation matrix $\mathbf{A}$ can be specified as $\mathbf{A}_{(1)} = \frac{1}{\sqrt{w_1^2+w_2^2}} \left(\begin{smallmatrix} w_1 & w_2 \\ w_2 & -w_1 \end{smallmatrix} \right)$. In analogy to Supporting Information A, we can show that the transformed statistics
$\mathbf{y} = (T_{12,\text{agonistic}}, T_{12,\text{antagonistic}})^T = \mathbf{A}_{(1)}\mathbf{x}$ with $\mathbf{x} = \begin{pmatrix} T_1 \\ T_2 \end{pmatrix}$ are independent.

$$\begin{aligned}
\mathbf{\Sigma_y} &= \mathbf{A}_{(1)}\mathbf{\Sigma_x}\mathbf{A}_{(1)}^T \\
&= \frac{1}{\sqrt{w_1^2+w_2^2}} \begin{pmatrix} w_1 & w_2 \\ w_2 & -w_1 \end{pmatrix} \begin{pmatrix} 1 & 0 \\ 0 & 1 \end{pmatrix} \begin{pmatrix} w_1 & w_2 \\ w_2 & -w_1 \end{pmatrix} \frac{1}{\sqrt{w_1^2+w_2^2}} \\
&= \frac{1}{w_1^2+w_2^2} \begin{pmatrix} w_1 & w_2 \\ w_2 & -w_1 \end{pmatrix} \begin{pmatrix} w_1 & w_2 \\ w_2 & -w_1 \end{pmatrix} \\
&= \begin{pmatrix} 1 & 0 \\ 0 & 1 \end{pmatrix}.
\end{aligned}$$

Alternatively, the transformation matrix $\mathbf{A}$ can be specified as $\mathbf{A}_{(2)} = \frac{1}{\sqrt{w_1^2+w_2^2}} \left(\begin{smallmatrix} w_2 & w_1 \\ w_1 & -w_2 \end{smallmatrix} \right)$.

The following results show simulation-based power analyses to compare ASSET with CCMA and both versions of wCCMA by using the same settings as mentioned in the main manuscript. Again, we obtained comparable results by setting equal baseline risks to both diseases (data not shown).

In almost all settings the Subset-based Meta-Analysis (ASSET) has slightly higher power to detect disease-specific, agonistic and antagonistic effects compared to CCMA and wCCMA. In the case of detecting disease-specific effects, the difference between the methods is smallest (Tables S2 and S3). The way the transformation matrix $\mathbf{A}$ is constructed for the wCCMA method increases the power of detecting either agonistic or antagonistic effects (Tables S2 and S3).
